# The effects of the first wave of the COVID-19 pandemic on the presentation of adolescents to acute mental health services in NHS Lanarkshire

**DOI:** 10.1192/bjo.2021.735

**Published:** 2021-06-18

**Authors:** Youstina Nagiub, Rekha Hegde

**Affiliations:** NHS Lanarkshire

## Abstract

**Aims:**

This project aimed to assess the effects of COVID-19 on the mental health of adolescents, reflected through their presentations to A&E departments in NHS Lanarkshire.

**Method:**

The psychiatry liaison database was searched for referrals of 17 year olds and under from April until August 2020.

All referrals to all acute hospital sites in Lanarkshire received from any source were included. The only exclusion criteria applied were age over 17 and unavailable assessment information.

The sources searched for information were: patient's electronic notes, Mental Health Assessment forms, Mental Health Risk Assessment forms and electronic letters. The following information was gathered:

- patient's age
- date, source and reason for referral
- hospital site of assessment
- outcome of assessment

**Result:**

- Between April and August 2020, the number of CAMHS A&E referrals increased every month except in July.
- The age range of CAMHS patients presenting to A&E were 12-17 years, with 17 being the most common age seen.
- 87% of referrals were from A&E.
- The two most common reasons for referrals were drug overdose and suicidal ideation.
- The most common outcome of assessment was a CAMHS referral.
- COVID-19 was a trigger for an adolescent's presentation to A&E in 31% of cases, the most common cause being struggling with the lockdown/restrictions.

**Conclusion:**

The mental health charity YoungMinds carried out several surveys throughout the COVID-19 pandemic's first wave. They demonstrated a detrimental effect on young people's mental health in the UK.

YoungMinds surveys revealed that 32% and 41% of young people experienced “much worse” mental health due to COVID-19. The findings of NHS Lanarkshire were similar, with 31% of adolescents presenting to A&E as a result of COVID-19.

No adolescent included in this review had contracted COVID-19 at any point. Their mental health was therefore impacted by the indirect effects of COVID-19 rather than the direct effects of infection. For the 31% of CAMHS presentations to A&E which were due to COVID-19, most young people struggled with the lockdown/restrictions.

The number of presentations to A&E increased every month between April and August 2020 except for July. This could be due to people's initial fear of coming to hospital and catching COVID-19. However, as infection and death rates decreased towards the summer, people may have regarded hospitals as safer. The general increase in referrals every month may also be a reflection of the worsening of young people's mental health the longer the pandemic extended.

